# Supplementary material for: Revealing New Landscape of Turbot (Scophthalmus maximus) Spleen Infected with Aeromonas salmonicida through Immune Related circRNA-miRNA-mRNA Axis
Source: Biology (Basel). 2021 Jul 6;10(7):626. doi: 10.3390/biology10070626 (PMC8301059; doi:10.3390/biology10070626)
Supplement: Supplementary file 1 [file biology-10-00626-s001.zip › biology-1237292-supplementary/Manuscript-supplementary/í∙Supplementnary Table 1 Primers used in this study.pdf]

Supplementntary Table 1 Primers used in this study.

| Primer name | Nucleotide sequence (5’-3’) | Purpose |
|---|---|---|
| SM novel_circ_0000498 F | AGAAAGAAGGCGAAGTGGACTC | qRT-PCR |
| SM novel_circ_0000498 R | TTGGAGGGAGACTCTGCGGTT | qRT-PCR |
| SM novel_circ_0004184 F | TCTCTGACTACAAGCACCACT | qRT-PCR |
| SM novel_circ_0004184 R | GATATGGCTTGAACACGATGGT | qRT-PCR |
| SM novel_circ_0002683 F | GGGTGCACTGTTGCATATACTT | qRT-PCR |
| SM novel_circ_0002683 R | CGGCTCACCTTGGATTTGAAC | qRT-PCR |
| SM novel_circ_0003361 F | GACTCCAGTGGCTGCTCTT | qRT-PCR |
| SM novel_circ_0003361 R | ACCAGAGCGATGAAGGTCC | qRT-PCR |
| SM dre-miR-223 | TGTCAGTTTGTCAAATACCCC | qRT-PCR |
| SM dre-miR-200a-3p | TAACACTGTCTGGTAACGATG | qRT-PCR |
| SM dre-miR-200b-3p | TAATACTGCCTGGTAATGATGA | qRT-PCR |
| SM dre-miR-122 | TGGAGTGTGACAATGGTGT | qRT-PCR |
| SM gene14346 F | CAGCCAGATCCAGCACATCAC | qRT-PCR |
| SM gene14346 R | AGGCGCTGAACTGAAACGAGT | qRT-PCR |
| SM gene658 F | GTTGCAGTGACACTCGTGCTC | qRT-PCR |
| SM gene658 R | ACGGTTCCATTGACGTCTCTT | qRT-PCR |
| SM gene9492 F | ATGGCGACTTCTTCCCAATCT | qRT-PCR |
| SM gene9492 R | GGCCTCTGTGGTCAGGTTG | qRT-PCR |
| SM gene1381 F | CTCATCAACACCTGTTACACCT | qRT-PCR |
| SM gene1381 R | TGCAAAGGCACAAATGATCATCAG | qRT-PCR |
| 18S qF | TGTGGGTTTTCTCTCTCTG | qRT-PCR |
| 18S qR | ATTCTTGGCAAATGCTTTC | qRT-PCR |
| U6 F | GCTTCGGCAGCACATATACTAAAAT | qRT-PCR |
| U6 R | CGCTTCACGAATTTGCGTGTCAT | qRT-PCR |
